# Insulin prices, availability and affordability: a cross-sectional survey of pharmacies in Hubei Province, China

**DOI:** 10.1186/s12913-017-2553-0

**Published:** 2017-08-24

**Authors:** Chenxi Liu, Xinping Zhang, Chaojie Liu, Margaret Ewen, Zinan Zhang, Guoqin Liu

**Affiliations:** 1 School of Medicine and Health Management, Tongji Medical School, Huazhong University of Science and Technology, No.13 Hangkong Rd, Wuhan, Hubei Province China; 2 China Health Program, La Trobe University, Plenty Road & Kingsbury Drive, Melbourne, VIC 3086 Australia; 3 Health Action International, Overtoom 60-2hg, Amsterdam, 1054 HK The Netherlands; 4 School of Management, Zunyi Medical University, No.201 Dalian Road, Zunyi, Guizhou Province China

**Keywords:** Access, Availability, Affordability, Price component, Insulin, Diabetes, China, WHO/HAI

## Abstract

**Background:**

Poor access to affordable insulin results in serious and needless complications and premature deaths for those with diabetes who need this essential medicine. To help address this issue, we assessed insulin availability, prices, affordability and price components in Hubei Province as China has the heaviest burden of diabetes globally.

**Methods:**

In 2016, insulin availability and price data was collected in the capital and five other cities. A total of 30 public sector outlets (hospitals and primary care institutions) and 30 private pharmacies were sampled, using an adaptation of the World Health Organization/Health Action International methodology, Data was collected for all human and analogue insulins in stock, then analyzed by type (prandial, basal or pre-mixed) and duration of action. Prices were expressed as Median Price Ratios (MPRs) to Australian PBS prices. Price components were tracked for five insulin products in two cities.. Affordability was assessed as the number of days’ wages of the lowest paid unskilled government worker needed to purchase 10 ml 100 IU/ml (approximately 30 days’ supply).

**Results:**

Mean availability was highest in public hospitals for prandial (70%), basal (80%) and pre-mixed insulin (90%). In primary care institutions and private pharmacies mean availability ranged from 10% to 33%. Median prices of all insulin types were higher that Australian PBS prices in all three sectors for human and analogue insulins (ranging from1.36–2.59 times). Patients have to pay 4 to 16 days’ wages to purchase a month’s treatment depending on the insulin type and sector. The largest component of the patient price was the manufacturers’ selling price (60%). Taxes in the form of import duties and VAT are applied in some sectors.

**Conclusions:**

The availability of insulin in primary care institutions and private retail pharmacies was very low in Hubei. Only public hospitals had good insulin availability. Insulin prices were high in all sectors making this life-saving medicine unaffordable, especially for those on low incomes. Governments should consider using its bargaining power to reduce prices, abolish taxes on essential medicines such as insulin, and develop strategies for more equitable access to insulin.

**Electronic supplementary material:**

The online version of this article (doi:10.1186/s12913-017-2553-0) contains supplementary material, which is available to authorized users.

## Background

Over the past few decades, non-communicable diseases (NCDs) have surpassed communicable diseases and become a major health challenge in the world [1]. Diabetes, one of the most prevalent NCDs, has since been prioritized in the NCDs strategy of the World Health Organization (WHO) because of its significant contribution to morbidity and mortality [2]. It was estimated that diabetes led to 1.5 million deaths in 2012 and was associated with more than 60% of all deaths [3, 4]. This has led the United Nations (UN) to publish global plans of actions to tackle diabetes and other NCDs [2, 5].

Effective control of blood glucose is crucial for mitigating the risks of morbidity and mortality associated with diabetes. The discovery of insulin in 1921 [6] ended the harsh approach of calorie management and a miserable death from starvation of diabetic patient [7]. Nowadays, the life expectancy of children in the USA with Type 1 Diabetes is only 4–6 years less than the general population [8].

However, poor access to affordable insulin has resulted in needless complications and premature deaths in many parts of the world [9]. The life expectancy of children born with Type 1 Diabetes in sub-Saharan Africa is as low as one year [10]. It is challenging to find Type 1 Diabetes patients living beyond 30 years with his disease in China [11]. Although insulin has been included in the WHO Model Essential Medicines List (EML) since 1977 [12], high prices are making it unaffordable in many countries, even in some high-income countries. For example, discontinuation of insulin use because of the high price was the main cause of diabetic ketoacidosis in an inner city setting in the USA. The UK’s National Health Service has also experienced a growing financial burden in supplying insulin [13].

China bears the heaviest burden of diabetes in the world [3, 14]. About 12% of adults (nearly 113.9 million) [15] have diabetes. Of these, 30% will eventually require insulin treatment [16]. The prevalence of children with Type 1 Diabetes is also increasing in China. A recent study in Shanghai estimated that the number of children with Type 1 Diabetes will increase six-fold by 2025 [17].

This study measured the price, availability and affordability of insulin in Hubei province in China, and insulin price components (mark-ups etc.) in the pharmaceutical supply chain.

## Methods

### Study setting

Hubei is located in central China, with a population of over 60 million. Compared with other provinces, Hubei has the highest incidence (4.6/100,000 per year from 1988 to 1996) of children with Type 1 Diabetes [18] and a relatively lower prevalence (8.26% vs an average of 12%) of adults with diabetes. The mortality of diabetes in Hubei has been increasing at an average annual rate of 2.1% since 1990 [19].

In China, patients can obtain insulin from hospitals, primary care institutions, and private pharmacies with a prescription from a doctor. Public hospitals and primary care institutions dominate the market because the majority of doctors are employed by these institutions and patients are encouraged to have prescriptions dispensed in these outlets. However, there is no gate keeping arrangement. Patients can seek medical attention from hospital outpatient clinics without referral from primary care providers. More than 95% of Chinese people are covered by social health insurance [20].

Despite near universal coverage of social health insurance in China, a high percentage of out-of-pocket payment is still required. On average, out-of-pocket payment comprises 72.35% of total health expenditure in China [21]. Furthermore, the insurance contributions from governments and employers (if any) usually fund inpatient care. Because insulin is mainly prescribed in outpatient clinics, the major financial burden falls on patients. To reduce this financial burden, the Chinese government introduced a maximum procurement price policy [22] for public institutions. In addition, a zero-mark-up policy [23] has applied to primary care facilities since 2012; this is expected to soon expand to public hospitals. There are no medicine price or mark-up regulations in the private sector [24].

### Design and sampling

From August to September 2016 we firstly conducted a survey of insulin availability and prices in public and private sector outlets using an adaptation of the WHO/Health Action International (HAI) methodology [25]. Secondly, as part of the Addressing the Challenges and Constraints of Insulin Sources and Supply (ACCISS) Study, we measured price components (mark-ups, taxes etc.) for five selected insulin products as a case study.

For the insulin availability and prices survey, the capital and 5 other cities were selected based on their size, geographic location and economic status: Wuhan (the capital city with a population of 9.79 million people); three medium-sized prefecture-level cities (with a population ranging from 1.05 to 5.50 million); two smaller-sized county-level cities (with populations of about 0.95 million).

Although WHO/HAI recommended that there should be 5 hospitals and 5 primary cares in each survey area, respectively, it could not be met in most cities in Hubei Province. Thus, we revised our sampling to consistent with the distribution of healthcare facilities in each survey area, namely, two hospitals and three primary care institutions in each large or medium-sized city; one hospital and four primary care institutions in each small-sized city.

In each city, the largest public hospital was identified as a “local anchor”. Then, four other public sector pharmacies (hospitals or primary cares) within 15-min of driving distance from the local anchor were selected. Finally, one private retail pharmacy closest to each of the selected public pharmacies was selected. This resulted in a sample size of 60 pharmacies: 10 public hospitals, 20 primary cares and 30 private pharmacies (Table 1).

**Table 1:** Number of outlets sampled and characteristics of participating cities

City name	Number of facilities in each city (N)			City characteristics		
	Hospitals	Primary care	Private pharmacies	City size	Economic status	Geographic location
Wuhan	2	3	5	Large (Capital)	High-income	East
Ezhou	2	3	5	Medium (Prefecture)	High-income	Southeast
Xiangyang	2	3	5	Medium (Prefecture)	Middle-income	North
Enshi	2	3	5	Medium (Prefecture)	Low-income	Southwest
Qianjiang	1	4	5	Small (County)	High-income	Central
Huangpi	1	4	5	Small (County)	Middle-income	Northeast
Total	10	20	30			

### Data collection

Following training, which included piloting data collection, two PhD candidates (CXL and ZNZ) visited the selected pharmacies in pairs and collected data. Pharmacies were contacted in advance, collecting information (name, address) and agreeing an appropriate time to conduct the survey. Each interviewee was given an insulated cup ($3.78) as a gift. Following data collection, the data were checked by the supervisor (XPZ) for completeness.

Data was recorded on all human and analogue insulin products in each outlet, including the brand name, type, strength [international unit (IU)/ml], presentation [vial, cartridge or prefilled pen], volume [ml], pack size, pack price and manufacturer. The insulins found were categorized into sub-types according to the onset, peak time, and duration of effects. The insulin products were grouped into prandial (short-acting and rapid-acting), basal (intermediate-acting and long-acting), and pre-mixed (Additional file 1: Table S1).

Animal insulin products were not included in this study because our pilot survey showed that they were rarely available in Hubei province.

As a case study, we conducted face-to-face or phone interviews to identify price components (mark-ups and other charges) for the five mostly commonly used insulin products. Buying and selling prices were recorded in the supply chain, starting from the pharmacy outlets and tracking back to the manufacturers/importers. A literature review was used to identify the most commonly used insulin. This resulted in tracking price components of two human insulins and three analogues. All were imported products (Table 2). The data was collected in the capital (Wuhan) and one county-level city (Huangpi). In each city, the largest public hospital and one private pharmacy participated in the price components case study.

**Table 2:** Insulin products selected for the pricing component survey

Brand name	Insulin type	Manufacturers	Presentation	Imported/locally produced
Novolin Mix 30	Human	Novo Nordisk	Cartridge	Imported
Novolin Mix50	Human	Novo Nordisk	Cartridge	Imported
Lantus	Analogue	Sanofi	Pen	Imported
Humalog	Analogue	Eli Lilly	Cartridge	Imported
Humalog 25	Analogue	Eli Lilly	Cartridge	Imported

### Data analysis

Availability was defined as the percentage of pharmacies where insulin products were available at the time of the survey. We compared availability of different subtypes of insulin products in different outlets using Fisher exact tests. The following ranges were used for describing availability [26]:

• < 30% very low.

• 30–49% low.

• 50–80% fairly high.

• > 80% high.

Prices were expressed as a Median Price Ratio (MPR) i.e. a ratio of the local price to a standard set of international reference prices (IRPs). Thus, MPRs describe how much greater or less the price of an insulin product is compared to the IRP. The WHO/HAI methodology recommends Management Sciences for Health’s *International Drug Price Indicator Guide* as the source of the international reference price (IRP). However, the Guide does not include prices for analogue insulins so was of limited value. Therefore, prices from the Australia Pharmaceutical Benefit Scheme (PBS) [27] were used as IRPs. PBS prices represented reimbursement prices paid by the Australian government. We standardized all prices (PBS and the prices collected in Hubei) to US$ for 1000 IU insulin then calculated the MPRs.

$$ \mathrm{MPR}=\frac{\mathrm{Selling}\  \mathrm{price}\  \mathrm{of}\ 1000\mathrm{IU}\ \mathrm{in}\mathrm{sulin}\  \mathrm{in}\  \mathrm{Hubei}\  \mathrm{in}\ \mathrm{US}\ \$}{\mathrm{Selling}\  \mathrm{price}\  \mathrm{of}\ 1000\mathrm{IU}\ \mathrm{in}\mathrm{sulin}\  \mathrm{Australian}\ \mathrm{PBS}\ \mathrm{in}\ \mathrm{US}\kern0.50em \$} $$

Generally, an MPR of 1 or less represents efficient procurement in the public hospitals and primary cares, while an MPR below 3 is interpreted as acceptable prices for the private sector [28, 29].

Affordability was expressed as the number of days the lowest paid unskilled government worker needs to work to purchase 1000 IU (approximately 30 days’ supply) of insulin based on average treatment costs [30]. In 2016, the average daily wage of unskilled government workers was US$6.40 in Hubei. We considered paying over one day’s wage for one month’s therapy is unaffordable [31].

We tested normality and homogeneity of variances of prices and affordability, and performed either parametric (independent *t* test or One-way ANOVA) or non-parametric (Kruskal–Wallis test or Mann-Whitney U test) analyses to compare the differences of those indicators across the pharmacies per sector.

All statistical analyses were conducted using SPSS 12.0 and a *p* value < 0.05 was considered as statistical significance.

For insulin price components, the add-on charges identified in each sector where grouped as follows: manufacturer’s selling price (MSP), import duty, importer’s mark-up, wholesaler’s mark-up, outlet’s mark-up and value-added tax (VAT).

## Results

Across the 60 pharmacies sampled, 186 price points were recorded. Of these, 90 were for human insulin and 96 were for analogues. All were presented in cartridges or prefilled pens containing 300 IU/3 ml. No insulin in vials were found. Most of the insulin products (81.18%) were imported (Table 3).

**Table 3:** Insulin products found in the pharmacies

Characteristics		Number of products
Type of insulin	Human insulin	
	Short-acting	14 (7.53%)
	Intermediate-acting	10 (5.38%)
	Mixed	66 (35.48%)
	Analogue insulin	
	Rapid-acting	23 (12.37%)
	Long-acting	28 (15.05%)
	Mixed	45 (24.19%)
Supplier	Imported	
	Novo Nordisk A/S	93 (50.00%)
	Eli Lilly Italia S.p.A./ Lilly France	41 (22.04%)
	Sanofi-Aventis Deutschland GmbH	12 (6.45%)
	BIOTON S.A.	5 (2.69%)
	Locally produced	
	Gan Lee Pharmaceutical co., Ltd.	7 (3.76%)
	Dongbao Pharmaceutical co., Ltd.	14 (7.53%)
	Lianbang Ppharmaceutical co., Ltd.	14 (7.53%)
Presentation	Cartridge, containing 300 IU/3 ml	155 (83.33%)
	Prefilled Pen, containing 300 IU/3 ml	31 (16.67%)

### Availability

Significant differences in availability were found for all insulin types across the outlets (*p* < 0.05), except for intermediate-acting human insulin products. The highest availability of insulin was found in public hospitals compared with primary care institutions and private pharmacies (Table 4).

**Table 4:** Mean availability (%) of insulin in outlets

Type of insulin		Public hospital	Primary care facility	Private retailer	*P* value*
Prandial insulin	Overall	70%	20%	13%	0.008
	Human short-acting	50%	10%	10%	0.018
	Analogue rapid-acting	70%	15%	10%	0.001
Basal insulin	Overall	80%	10%	20%	< 0.001
	Human intermediate-acting	20%	0%	17%	1.000
	Analogue long-acting	70%	10%	13%	0.001
Pre-mixed insulin	Overall	90%	20%	33%	0.001
	Human	90%	15%	27%	< 0.001
	Analogue	80%	15%	20%	0.001

*p value of Fisher’s exacts

Overall, 90% of public hospitals had pre-mixed insulin products (90% human and 80% analogue). Although the availability of short-acting (50%) and intermediate-acting (20%) human insulin was low, 70% of public hospitals had rapid-acting and long-acting analogue insulin (Table 4).

Insulin availability in primary care institutions was very low (10% to 20%). No intermediate-acting human insulin was available in primary care institutions. Overall, the availability of insulin in private pharmacies ranged from 13% for prandial (10% short-acting and 10% rapid-acting) to 33% for pre-mixed (27% human and 20% analogue). Consistent with public hospitals, primary care institutions and private pharmacies were more likely to have pre-mixed insulin products than others (Table 4).

### Patient prices and affordability

Prices of insulin products were higher than Australian PBS prices, with median MPRs greater than 1 for all insulin types. Between 4 to 16 days’ wages of the lowest paid unskilled government worker is needed to purchase a month’s treatment depending on the insulin type purchases and the sector (Table 5).

**Table 5:** Median Price Ratio (MPR) and average affordability of insulins in different kind of outlets (Mean ± Standard)

Type of insulin		Indicators	Public hospital	Primary care institution	Private retailer	Overall	*p* value*
Prandial insulin	Human short-acting	MPR	1.41 ± 0.12	1.37 ± 0.13	1.52 ± 0.13	1.44 ± 0.13^†^	0.173
		Affordability	4.35 ± 0.37	4.24 ± 0.41	4.68 ± 0.40	4.45 ± 0.40^†^	
	Analogs rapid-acting	MPR	1.69 ± 0.14	1.67 ± 0.13	1.76 ± 0.32	1.70 ± 0.19	0.957
		Affordability	6.29 ± 0.53	6.19 ± 0.50	6.53 ± 1.20	6.33 ± 0.69	
Basal insulin	Human intermediate-acting	MPR	1.49 ± 0.11	Not Available	1.57 ± 0.24	1.55 ± 0.21^†^	0.636
		Affordability	4.61 ± 0.35	Not Available	4.84 ± 0.75	4.77 ± 0.43^†^	
	Analogs long-acting	MPR	2.59 ± 0.33	2.23 ± 0.24	2.53 ± 0.27	2.53 ± 0.32	0.155
		Affordability	16.11 ± 1.89	14.16 ± 1.53	15.72 ± 1.61	15.8 ± 01.84	
Pre-mixed insulin	Human	MPR	1.49 ± 0.40	1.28 ± 0.20	1.36 ± 0.29	1.43 ± 0.36^†^	0.018
		Affordability	4.61 ± 1.23	3.95 ± 0.61	4.19 ± 0.90	4.43 ± 1.11^†^	
	analogs	MPR	1.59 ± 0.20	1.59 ± 0.23	1.68 ± 0.21	1.62 ± 0..21	0.401
		Affordability	5.92 ± 0.75	5.91 ± 0.87	6.26 ± 0.79	6.02 ± 0.76	

*t tests or ANOVA for data with normal distribution; Kruskal Wallis tests or Mann-Whitney U tests for data with non-normal distribution

^†^ *p* < 0.001 compared with analogs products

Overall, analogue insulins were higher priced and less affordable than human insulins (*p* < 0.001). Long-acting analogue insulins were highest priced, with median MPRs ranging from 2.23 to 2.59 compared with about 1.5 for the other insulin products. Overall, across the sectors, 14–16 days wages are needed to purchase long-acting analogues, compared to 4–7 days for other insulins.

Insulin products dispensed from primary care institutions were lower priced and more affordable than those dispensed from public hospitals and private pharmacies. However, such differences were only statistically significant for pre-mixed human insulin (*p* = 0.018).

### Insulin price components

For each of the five insulin brands (2 human, 3 analogues) in the analysis, price components were categorized into four stages namely, MSP, importer’s mark-up, wholesaler’s mark-up and outlet’s mark-up (Actual prices and contribution from stakeholders were showed in Additional file 2: Table S2). After summarized, the main contribution of price (over 60%) was the MSP for all five brands. In accordance with Chinese tax policy, import duty was levied on importers and VAT was levied on importers, wholesalers and private retailers. Public hospitals were tax exempt. The VAT was extracted from importers, wholesalers and private retailers then summarize based on tax policy, patients are required to pay VAT on the retail price.

**Fig. 1:**
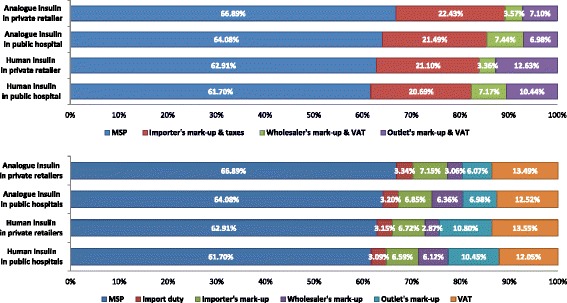
Contribution of price components to the final patient price of insulin. The first histogram indicated the original stakeholders’ contribution (with taxes) to the final prices. To show the impact of tax, import duty and value added tax was extracted and the second histogram showed the mark-ups of different stakeholders (without tax) and cumulative contribution of taxes

Figure 1 shows the contribution of price components to the final patient price, for one human insulin and one analogue in the two sectors. In the top four bars, the mark-ups include VAT. In the second set of bars, VAT was extracted from the mark-ups and added to the VAT paid by patients. In total VAT contributed 12.05% to 13.55% to final prices and import duty contributed about 3%. Cumulatively, taxes constituted over 15% to final insulin prices. Excluding VAT, importers’ mark-ups contributed about 7% in both sectors for human and analogue insulin. The contribution of the wholesaler’s mark-up doubled when insulin was sold to public hospitals (6% vs 3%). The contribution of private retail and hospital mark-ups for analogues were 6–7%, while the contribution for human insulin was about 10% in both sectors.

## Discussion

To our knowledge, this is the first study of its kind in China to analyze the availability, price, affordability and price components of insulin in the public and private sectors. We found that the availability of pre-mixed insulin products was good in public hospitals. But the availability of insulin in primary care institutions and private pharmacies was sub-optimal. A study in 15 developing countries revealed that insulin was available in 56% (17–100%) of public facilities and 39% (0–95%) private facilities [32].

The pricing level of insulin products is high, leading to poor affordability. This is particularly the case for long-acting analogue insulin, which costs half of a month wage of the lowest paid government worker for a month’s treatment. About 60% of the price was due to the MSP, with taxes and mark-ups also important contributors.

### Availability of insulin

A wide range of insulin products need to be made available in pharmacies. This is because insulin treatment is highly individualized due to variations of lifestyle, calories intake, and responsiveness of patients. The American Diabetes Association recommends combined use of different insulin products for children with Type 1 Diabetes and in adults whose blood glucose level is difficult to control [33]. Our study shows that only public hospitals are likely to be able to meet this recommendation for insulin treatment.

Although patients can seek medical attention from any facility without referral, buying insulin from a public hospital is more inconvenient and time consuming than visits to primary care institutions and private pharmacies. Unfortunately, the availability of insulin products in primary health centres was low although they are slightly more affordable. Therefore patients have to visit public hospitals frequently to obtain insulin supplies. This would disadvantage those who live far away from hospitals, the elderly, children and patients who have amputations with diabetic feet. The inconvenience and additional travel costs could lead to disrupted treatment and poor management of blood glucose, resulting in serious complications.

### Affordability of insulin

Overall, the pricing level of insulin products is high, leading to poor affordability of insulin. A month’s treatment costs at least four days’ wages, even in the public sector. In reality, the affordability of insulin may be even worse because diabetes is associated with poverty and many diabetic patients earn a lower wage than the lowest paid unskilled government workers [34].

The high pricing level is also likely to impose high financial burden on patients [30]. Although China has achieved almost universal coverage of health insurance, insurance benefits are limited. High percentages of out-of-pocket payments are required for medicines [20]. A national survey showed that less than 14% of direct costs of medicines dispensed through outpatient outlets are covered by those insurance programs [35].

### Pricing components

Identifying price components provides insight into why insulin prices may be high and potential of cost reduction. The MSP accounts for over 60% of the final patient price. It should be reduced given the large number of diabetic patients in China [15]. But only the government has the bargaining power due to the lack of highly functioning non-governmental bodies. Unfortunately, the Chinese government has recently abandoned an initiative that established medicine prices based on scientific evidence and negotiations with the pharmaceutical industry. The medicine price setting systems in China are currently decentralized and fragmented, jeopardizing the bargaining power of the government. Each provincial government set its own pricing policy for public hospitals and primary care institutions [22]. It is also important to note that the Chinese market has few insulin manufacturers, which may impose additional barriers on the cost reduction efforts of governments [30, 36].

Abolishing taxes can reduce the price of medicines. VAT contributes 12% to 14% of the total patient price of insulin. Experience in some developing countries [30] shows that insulin price can be reduced by about 15% if government tax is eliminated. Considering the fact that these tax levies are imposed on the sick and are inequitable, and most diabetic patients are children (Type 1 Diabetes) and elderly (Type 2 diabetes), there is a strong rationale to exempt insulin from VAT.

The current government policy has a strong focus on reducing mark-ups of public health providers when dispensing medicines. This is understandable because most medicines are dispensed through public providers. In the past, the mark-up policy (15%) had encouraged doctors to prescribe high priced medicines to maximise profits for their organizations [37]. Since 2012, China has initiated a zero-mark-up policy for pharmaceutical products in public health care institutions [23]. This study found that the mark-up for dispensing insulin comprised 6% to 11% of final patient price of insulin.

Insulin supplied in vials is needed as they tend to be cheaper [38], while pens/cartridges offer more options for patients who can afford them. The disappearance of vials may tie patients to insulin injection devices, some of which are patented, and the associated cartridges [36]. However, insulin in vials appears to no longer be available in the Hubei market, although it is still registered with the National Food and Drug Administration [39]. The WHO advises that human insulin in vials should be available in outlets to meet the basic demands of patients [2]. However, the transition from vial to cartridge/pen has been observed all over the world [30]. The reasons behind the transition are still not fully understood. It may be a combination of many factors, including less wastage, better adherence and higher quality of life [40], only if patients could afford them.

### Policy implications

China is attempting to establish a primary care dominated tiered health care system. Such a system is supposed to be able to provide more cost-efficient and cost-effective services to consumers [41]. Patients, especially those with chronic conditions, are encouraged to obtain more services from primary care centres than from hospitals. However, appropriate care for people needing insulin cannot be provided in primary care facilities unless they are equipped with needed insulin products at affordable prices. Singular government subsidies through insurance programs are not enough to alleviate the financial burden of accessing insulin. Prices of insulin products are too high in China; the government needs to use its bargaining power to reduce the price of insulin and make it more affordable, especially for the poor.

### Limitations

This study has several limitations. Firstly, the study was conducted in Hubei province and hence may not be representative of all of China. Secondly, a few private pharmacies did not keep insulin in stock but could order for their customer on request. Therefore, the availability of insulin in private pharmacies is likely to be underestimated. Thirdly, the IRPs were from PBS of Australia, rather than International Drug Price Indicator Guide which was recommended by WHO/HAI, in current study. The MPR results can be skewed by a particularly high or low price from PBS. Finally, MSP and importers’ mark-ups were estimated, rather than self-reported. The importers mark-up includes profit as well as transport costs. The importers’ mark-up was based on wholesaler mark-ups because delivery costs of importers are similar to those of wholesalers. The MSP was back calculated based on importers’ mark-ups.

## Conclusion

This study provides evidence on access to insulin problems in Hubei. The availability of insulin in primary care institutions and private retail pharmacies was very low. Only public hospitals had good insulin availability. However, in hospitals, primary care institutions and private retail pharmacies, insulin prices were high making this life-saving medicine unaffordable, especially for those on low incomes. The government should consider using its bargaining power to reduce prices, abolish taxes on essential medicines such as insulin, and develop strategies for more equitable access to insulin.

## Additional files

Additional file 1: Table S1. Categorization of insulin products. The Table S1 showed the categorization of insulin products applied in current study, in which the insulin products were grouped into prandial (short-acting and rapid-acting), basal (intermediate-acting and long-acting), and pre-mixed. (DOCX 16 kb)

Additional file 2: Table S2. Mark-ups in the supply chain for 5 tracked insulin products (Ұ). The Table S2 showed the actual prices and contribution from different stakeholders for each of the five insulin brands (2 human, 3 analogues) in the analysis. The price components were categorized into four stages namely, MSP, importer’s mark-up, wholesaler’s mark-up and outlet’s mark-up according to the methodology of HAI/WHO. (DOCX 15 kb)

## Abbreviations

EML: Essential Medicine List
HAI: Health Action International
IRP: International Reference Price
MPR: Median Price Ratio
MSP: Manufacturer’s Selling Price
NCD: Non-communication disease
PBS: Pharmaceutical Benefit Scheme
UN: United Nations
VAT: Value added tax
WHO: World Health Organization
