# Supplementary material for: Insulin prices, availability and affordability: a cross-sectional survey of pharmacies in Hubei Province, China
Source: BMC Health Serv Res. 2017 Aug 24;17:597. doi: 10.1186/s12913-017-2553-0 (PMC5571633; doi:10.1186/s12913-017-2553-0)
Supplement: Supplementary file 1 — Categorization of insulin products. The Table S1 showed the categorization of insulin products applied in current study, in which the insulin products were grouped into prandial (short-acting and rapid-acting), basal (intermediate-acting and long-acting), and pre-mixed. (DOCX 16 kb) [file 12913_2017_2553_MOESM1_ESM.docx]

|  | **Table S1. Categorization of insulin products** | | |
| --- | --- | --- | --- |
| **Types of insulin** | | **Ingredient** | **Brand name** |
| Prandial insulin | Human short-acting | Regular insulin | Novolin R; Humulin R;  Gansulin R; SciLin R |
|  | Analogue rapid-acting | Aspart insulin | NovoRapid Penfill;  NovoRapid Flexpen |
|  |  | Lispro insulin | Humanlog |
| Basal inulin | Human intermediate-acting | Isophane insulin | Novolin N Flexpen; Novolin N Penfill;  USLIN N; Ganshulin N |
|  | Analogue long-acting | Glargine insulin | Lantus SoloStar;  Chang Xiu Lin Glargine |
|  |  | Determir insulin | Levemir Penfill;  Levemir Flexpen |
| Pre-mixed insulin | Human pre-mixed | Isophane/regular insulin | Humulin 70/30; Novolin 30R Penfill;  Novolin 50R Penfill; Ganshulin 30R  Scilin M30 (30/70); USLIN 30R (30/70);  USLIN 50R (50/50) |
|  | Analogue pre-mixed | Aspart/protamine insulin | Novo Mix 30 Penfill; Novo Mix 30 Flexpen;  Novo Mix 50 Penfill; Novo Mix 50 Flexpen |
|  |  | Lispro/protamine insulin | Humalog Mix 25  Humalog Mix 50 |
