# Supplementary material for: Insulin prices, availability and affordability: a cross-sectional survey of pharmacies in Hubei Province, China
Source: BMC Health Serv Res. 2017 Aug 24;17:597. doi: 10.1186/s12913-017-2553-0 (PMC5571633; doi:10.1186/s12913-017-2553-0)
Supplement: Supplementary file 2 — Mark-ups in the supply chain for 5 tracked insulin products (Ұ). The Table S2 showed the actual prices and contribution from different stakeholders for each of the five insulin brands (2 human, 3 analogues) in the analysis. The price components were categorized into four stages namely, MSP, importer’s mark-up, wholesaler’s mark-up and outlet’s mark-up according to the methodology of HAI/WHO. (DOCX 15 kb) [file 12913_2017_2553_MOESM2_ESM.docx]

**Table S2. Mark-ups in the supply chain for 5 tracked insulin products (￥)**

| Outlet | Region | Medicine Name | Dosage Form | Manufacturer | Manufacturer's selling price* | Importers’ mark-up including import duty and VAT* | Wholesalers’ mark-up including VAT | Outlets’ mark-up including VAT | Final price |
| --- | --- | --- | --- | --- | --- | --- | --- | --- | --- |
| Public hospital | Wuhan | Novolin 30 | Cartridge | Novo nordisk | 35.67 | 11.96 | 4.15 | 12.42 | 64.20 |
| Public hospital | Wuhan | Lantus Solostar | Pen | Sanofi | 141.98 | 47.61 | 16.49 | 30.92 | 237.00 |
| Public hospital | Wuhan | Humalog | Cartridge | Eli Lilly | 51.03 | 17.12 | 5.93 | 11.12 | 85.20 |
| Public hospital | Wuhan | Humalog 25 | Cartridge | Eli Lilly | 51.03 | 17.12 | 5.93 | 11.12 | 85.20 |
| Private retailer | Wuhan | Novolin 30 | Cartridge | Novo nordisk | 35.67 | 11.96 | 1.90 | 10.27 | 59.80 |
| Private retailer | Wuhan | Novolin 50 | Cartridge | Novo nordisk | 38.47 | 12.9 | 2.05 | 6.38 | 59.80 |
| Private retailer | Wuhan | Lantus Solostar | Pen | Sanofi | 142.60 | 47.83 | 7.62 | 11.95 | 210.00 |
| Private retailer | Wuhan | Humalog | Cartridge | Eli Lilly | 51.04 | 17.11 | 2.73 | 8.12 | 79.00 |
| Private retailer | Wuhan | Humalog 25 | Cartridge | Eli Lilly | 51.03 | 17.12 | 2.73 | 8.12 | 79.00 |
| Public hospital | Huangpi | Novolin 30 | Cartridge | Novo nordisk | 35.67 | 11.96 | 4.14 | 4.04 | 55.81 |
| Public hospital | Huangpi | Novolin 50 | Cartridge | Novo nordisk | 37.16 | 12.46 | 4.32 | 1.90 | 55.84 |
| Public hospital | Huangpi | Lantus Solostar | Pen | Sanofi | 141.98 | 47.61 | 16.49 | 0.00 | 206.08 |
| Public hospital | Huangpi | Humalog | Cartridge | Eli Lilly | 51.03 | 17.12 | 5.93 | 0.00 | 74.08 |
| Public hospital | Huangpi | Humalog 25 | Cartridge | Eli Lilly | 51.03 | 17.12 | 5.93 | 0.00 | 74.08 |
| Private retail | Huangpi | Novolin 30 | Cartridge | Novo nordisk | 35.67 | 11.96 | 1.90 | 6.47 | 56.00 |
| Private retail | Huangpi | Novolin 50 | Cartridge | Novo nordisk | 37.16 | 12.46 | 1.99 | 6.39 | 58.00 |
| Private retail | Huangpi | Humalog | Cartridge | Eli Lilly | 51.03 | 17.12 | 2.73 | 3.22 | 74.10 |

*Estimated
